# What to Choose Next? A Paradigm for Testing Human Sequential Decision Making

**DOI:** 10.3389/fpsyg.2017.00312

**Published:** 2017-03-07

**Authors:** Elisa M. Tartaglia, Aaron M. Clarke, Michael H. Herzog

**Affiliations:** ^1^Laboratory of Psychophysics, Brain Mind Institute, École Polytechnique Fédérale de Lausanne (EPFL)Lausanne, Switzerland; ^2^Aging in Vision and Action Lab, Sorbonne Universités, UPMC Univ Paris 06, INSERM, CNRS, Institut de la VisionParis, France; ^3^Psychology Department and Neuroscience Department, Aysel Sabuncu Brain Research Center, Bilkent UniversityAnkara, Turkey

**Keywords:** reinforcement learning, exploration, SARSA(λ), Q-learning, sequential decision making

## Abstract

Many of the decisions we make in our everyday lives are sequential and entail sparse rewards. While sequential decision-making has been extensively investigated in theory (e.g., by reinforcement learning models) there is no systematic experimental paradigm to test it. Here, we developed such a paradigm and investigated key components of reinforcement learning models: the eligibility trace (i.e., the memory trace of previous decision steps), the external reward, and the ability to exploit the statistics of the environment's structure (model-free vs. model-based mechanisms). We show that the eligibility trace decays not with sheer time, but rather with the number of discrete decision steps made by the participants. We further show that, unexpectedly, neither monetary rewards nor the environment's spatial regularity significantly modulate behavioral performance. Finally, we found that model-free learning algorithms describe human performance better than model-based algorithms.

## Introduction

Everyday actions are usually not recompensed by immediate reward. We have to make sequences of decisions before reaching a goal and sometimes a decision can pull us away from the goal, rather than getting us closer to it. Imagine developing a recipe for a new kind of cake. You start by adding ingredient after ingredient to the dough, but, you will not know whether you added too much or too little yeast until your cake is out of the oven. In this case, the feedback is not only delayed, but also sparse, making it difficult to infer each action's outcome. These situations are usually referred to as *sequential decision-making*.

There are a plethora of sequential decision making models, most of them relying on reinforcement learning algorithms (RL), such as SARSA(λ) or Q-learning, which can solve these types of problems (for a full exposition of learning models see Sutton and Barto, 1998; Barraclough et al., 2004; Gold and Shadlen, 2007; Dayan and Daw, 2008; Furman and Wang, 2008; McKinstry et al., 2008; Resulaj et al., 2009; Cisek and Kalaska, 2010; Solway and Botvinick, 2012; Dolan and Dayan, 2013). In RL models, it is assumed that an agent is in one of a number of discrete states. In each state *s*, the agent chooses an action *a* that brings the agent to a new state *s'* until a goal state is reached, a *reward* is collected, and an *episode* is completed. For example, an agent moving on a checker board can go north, south, west, or east. For each state *s*, the agent estimates the mean future reward when choosing action *a*, and this reward's value is denoted *Q*(*s*,*a*).

The learning objective of the model is to choose the state/action pairs that maximize reward collection. When a goal is found, the obtained reward is compared with the expected reward, that is, the reward calculated by taking the difference between the *Q*-values at the rewarded and the previous locations. The *Q*-value at the rewarded location is adjusted based on how much the observed reward differs from the expected reward. In addition, some of the *Q*-values for the states and actions leading up to this penultimate state are also updated, indicating that they lead to a reward, that is, there are a number of state-action pairs that are eligible for estimate updates (this is called the “eligibility trace” method). In *model-based* learning, the agent makes explicit use of the learned probability transitions between states and/or of reward contingencies. Simply put, the agent forms an explicit map of the environment and simulates various actions before taking an actual step. Model-based learning is particularly useful in navigation tasks, in which the buildup of an internal representation of the environment allows for efficient planning of the sequence of right/left turns needed to reach a final destination. In *model-free* learning, the agent does not build up a model of the environment; it just updates *Q*-values, which tell the agent which actions are most likely to yield reward at which states, but not how the states are related to each other through those actions (Sutton and Barto, 1998).

**Figure 1:**
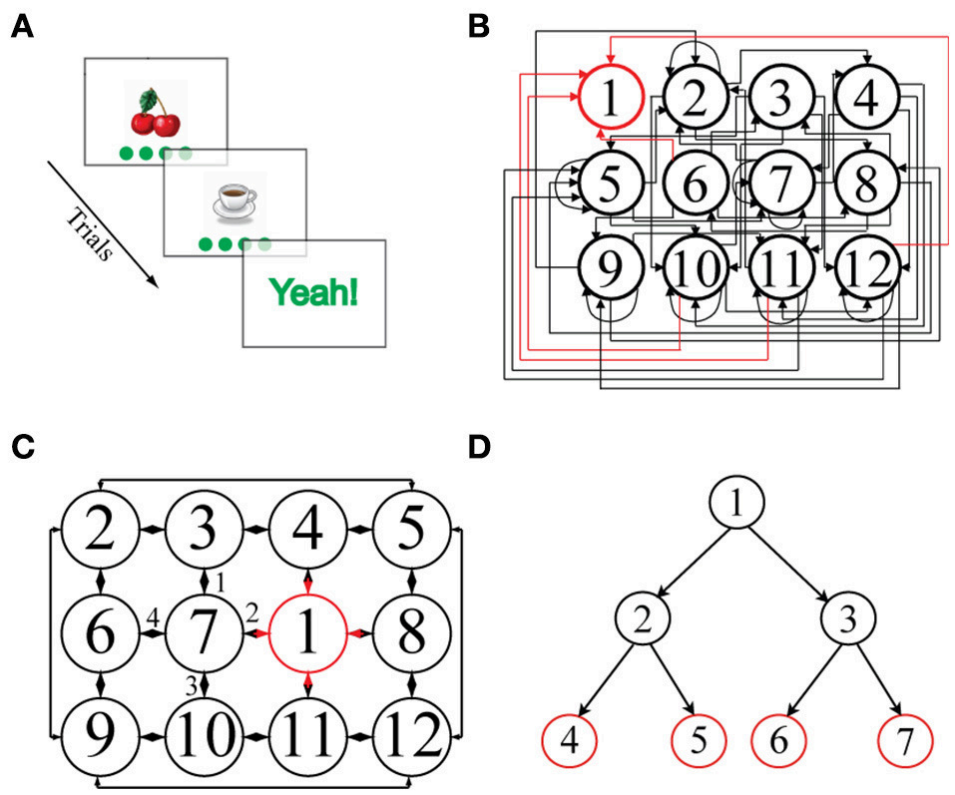
**(A)** Schematic representation of a single episode. An image, here “cherries,” is presented together with four green disks representing the potential actions. Clicking on one of the disks leads to the next image. **(B)** Illustration of a 2D-*non-embeddable* environment, defined by the state-action transition matrix shown. Each node represents a state and is associated with a unique image. State 1 (outlined in red) is the goal state and is always associated with the image “Yeah!.” Most of the actions connect non-neighboring states and are unidirectional: only rarely are direct back and forth transitions possible. Self-referential actions are frequent, i.e., an action often leaves the state unchanged. **(C)** Illustration of a 2D-*embeddable* environment. Actions connect neighboring states bi-directionally, e.g., if an action goes from “cherries” to “coffee cup,” there exists an inverse action that goes from “coffee cup” back to “cherries.” There are no self-referential (recurrent) actions, i.e., every action necessarily leads to a different state. Hence, in a 2D-embeddable environment, states and actions are arranged in a *grid-world* structure. **(D)** Illustration of a tree-search structure, typically used in other sequential decision making tasks present in the literature, in which multiple goal states (in red) can be reached after a fixed number of decision steps (i.e., two consecutive actions in the depicted example).

Current paradigms for investigating sequential decision making in human participants rely on tree-search environment structures, in which a few consecutive binary choices have to be made to accumulate evidence about the rewards at one of multiple goal states (Daw et al., 2005, 2011; Gläscher et al., 2010; Huys et al., 2012, 2015; Wunderlich et al., 2012; Solway and Botvinick, 2015). In these paradigms, nodes in the decision tree represents states and branches departing from each node represent available actions. From each node only two successor nodes can be reached (i.e., there are two available actions at each state); tree branches are independent so that each sequence of actions leads to a unique goal; actions inevitably bring the agent closer to, never away from, one of the final goal states and, most importantly, all participants attain one of the final goal states after the same, fixed number of actions, i.e., of decision steps (Figure 1D). Here, we have developed a more complex environment structure to flexibly examine sequential decision making in a setting which more closely resembles everyday life situations. In our paradigm, each node is connected to up to four successor nodes, tree branches are interconnected, actions can bring the agent either closer to or farther away from a unique goal state and, most importantly, the goal state is reached after a variable number of decision steps, depending on the participant' ability to find the shortest path to the goal (Figures 1B,C).

To test reinforcement learning models, we leveraged our new paradigm in three *sequential decision making* experiments in which we independently manipulated critical RL model variables like the structure of the environment space (to test for the building-up of an internal model of the environment), the inter-stimulus-interval (ISI) between images (to test for the eligibility trace), and the conditions of reward delivery.

## General setup

Experiments were conducted on a Phillips 201B4 monitor, running at a screen resolution of 1,024 × 768 pixels and a refresh rate of 100 Hz, using a 2.8 GHz Intel Pentium 4 processor workstation running Windows XP. Experiments were scripted in Matlab® 7.11 using custom software and extensions from the Psychophysics Toolbox for Windows XP (Brainard, 1997; Pelli, 1997).

### Participants

A total of 68 naïve students from the École Polytechnique Fédérale de Lausanne (EPFL), the Université de Lausanne (UNIL), and the Beritashvili Institute of Physiology in Tbilisi participated in the experiments (age range 18–30). Students were paid by the hour for their participation (except for Experiment 3 where they were paid based on performance). This study was carried out in accordance with the recommendations of the “Commission cantonale d'étique de la recherche sur l'être humain,” Protocol 384/2011 with written informed consent from all subjects. All subjects gave written informed consent in accordance with the Declaration of Helsinki. The protocol was approved by the “Commission cantonale d'étique de la recherche sur l'être humain.” Participants were told they could quit the experiment at any time they wished.

### Stimuli and general procedure

The general layout for an experiment is illustrated in Figure 1A. Participants were presented with different images, i.e., *states*, and for each state they chose one out of four actions. Each action took the observer to a new image. The objective was to find the state-action sequence leading to the goal-state, which always came with a *reward*. The way the actions connect the states defines the *environment space*. The environment space and the reward delivery were deterministic: at a given state a certain action always led to the same next state and finding the goal-state always entailed a reward. Below, the stimuli and procedures are described in detail.

Stimuli consisted of clip-art images presented centrally on the screen (Figure 1A). Image size was approximately one quarter of the screen. The images were semantically irrelevant for the task. Before the first experiment, participants were presented with a screen showing the entire image set used in the experiment. The observation distance was ~50 cm. A 3 cm diameter disk was presented at the screen's bottom. Participants had to click on the disk using the mouse to start the experiment.

One image was presented per trial. For every image, participants could choose among four possible actions. The actions were represented by four gray, 3 cm diameter disks presented in a horizontal line at the screen's bottom as depicted in Figure 1A. The four gray disks appeared at the same time as the image. To avoid any possible confusion, each time the mouse cursor hovered over a given disk, the disk turned from gray to green. The stimulus remained on the screen until the observer made his or her choice by clicking on one of the disks. Immediately after the observer clicked on a disk, both the image and the disks disappeared. The next image appeared after a certain inter stimulus interval (ISI), i.e., after a blank screen. For a given image, the same action always led to the same next image. This is because the structure of the environment space (Figures 1B,C), which defines all the *image-action-image* transitions, was fixed at the beginning of each experimental condition and remained unchanged thereafter. The sequence of images (and disks) proceeded until the observer reached the *goal-state*. The goal-state was a flashing “Yeah!” positioned centrally on the screen for ~1 s. We refer to the sequence of states and actions leading up to and including the goal-state as an *episode*. Participants were instructed *ab initio* about the nature of the goal state, i.e., they knew what it looked like and that its position within the environment space remained unchanged across episodes. The participants' task was to reach the goal-state as frequently as possible within the allotted time (i.e., 15 min in Experiments 1 and 3; in Experiments 2a and 2b the allotted time varied depending on the ISI).

## Data analysis

We measured performance with three metrics.

### Measure I: number of completed episodes

First, performance was quantified by counting the number of episodes completed in the allotted time: the higher the number of episodes, the better the performance (note that each episode is composed of a given number of trials, i.e., the number of actions chosen by the participants until the goal was reached). Different participants have different reaction times and, thus, some could complete more episodes purely by means of their faster trial-by-trial reaction times and not necessarily because of faster learning. To compensate for this unwanted source of variability, first, we determined—in each condition—the minimum number of total trials across all participants, i.e., by summing up for each observer the number of trials over all episodes. For each observer, we used only this minimum number of trials and examined how many episodes they completed, i.e., we discarded all trials beyond the minimum.

### Measure II: path length

Second, to capture how learning evolved on an episode-by-episode scale, we computed the *path length*, i.e., the number of states visited before reaching the goal state in each episode. We re-referenced the path length in each episode to the optimal path length, i.e., we subtracted from the actual path length the minimum number of images needed to reach the goal-state and added one. Hence, a path length equal to one indicates that the observer reached the goal-state by taking a shortest path. Since participants' data were linear in log-log coordinates, we took the natural logarithm of the participants' path lengths (*PL*) and episode numbers (*E*) and fitted a linear function (which gave a good approximation to the data):

(1) ln(PL)=β×ln(E) + η

where β reflects the learning rate so that lower β values indicate better performance, i.e., participants learned faster to take shorter paths (β is negative). η indicates the initial performance, so that the higher η, the worse is the initial performance, i.e., the higher is the path length in the first few episodes. We computed fits for the individual participants and compared the averaged parameters, β and η, between conditions in a repeated-measures ANOVA. In all conditions, β or η were not significantly correlated with the observer's reaction times (results not shown). This indicates that our path length measure is independent of reaction times and allows us to use all of the existing data instead of cutting off the number of completed trials at the group minimum as in our *Number of Completed Episodes* analysis. Moreover, both the initial performance and the learning rate are independent measures from the number of episodes completed. Performance can be very bad at the beginning, e.g., if it takes a long time to find the goal during the first episode (high η), but then it could improve very quickly, i.e., low β, or, to the contrary, performance can be quite good from the beginning, i.e., low η, but decrease more slowly toward the optimal path length, i.e., high β. Therefore, even if both cases could theoretically yield an identical number of episodes completed, they would have completely different path-length diagrams.

### Measure III: exploratory behavior

Third, we quantified how exploratory participants were by plotting the number of different actions the participants tried over the course of all episodes in each condition, averaged across states and across participants. Higher numbers of actions chosen per state indicate more exploratory behavior (four is the maximum, i.e., all actions available in a given state have been taken at least once, and zero is the minimum). A repeated-measures ANOVA was conducted for each experiment with *average number of actions chosen per state* as the dependent variable and *experimental condition* as the independent variable.

Similarly to path length, the average number of actions chosen per state was not significantly correlated with the observer's reaction times in any condition, indicating that the participants' exploratory behavior was independent of reaction time.

## Modeling

We fitted observer's data individually to the standard reinforcement learning models Sarsa(λ) and Dyna-Q. In addition, we employed a simplified single-parameter model, which we call the exploration/exploitation model, which leverages the fact that the state-action transitions are deterministic in our experiments. The model memorizes the shortest deterministic paths to the goal from each visited state. Furthermore, the model uses a probabilistic policy, which sets the trade-off between participants' exploration and exploitation strategies in a slightly modified version of the classical epsilon-greedy strategy (Sutton and Barto, 1998). At each state the model chooses between exploring new paths that might lead to the goal (with probability p_explore_) or exploiting known paths to the goal (with probability p_exploit_ = 1-p_explore_), as in the epsilon-greedy strategy. However, in contrast to the epsilon-greedy strategy, as soon as all actions have been tried at a given state, p_explore_ is set to zero, and from then on, the model always exploits. The advantage of exploiting known paths to the goal in a deterministic environment (i.e., an environment in which state-action transitions do not stochastically change) is that once one has found the optimal path to the goal, there is no point in exploring any further paths.

**Figure 2:**
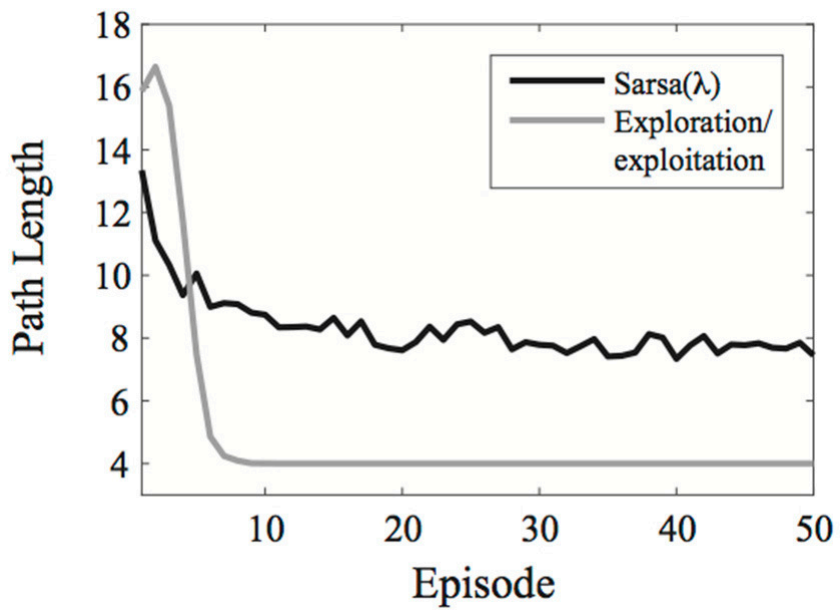
**Comparison of the exploration/exploitation model with the Sarsa(λ) model for the learning environment used in Experiment 1**. The exploration/exploitation algorithm quickly converges on the optimal path to the goal and continues exploiting it, while the Sarsa(λ) performs well initially, but takes much longer to converge on the optimal path to the goal, even when using an ε-greedy action selection rule. Here, each line represent averages over 500 simulated experiments. Plots show the best possible performance for each algorithm over the entire parameter space [three parameters for Sarsa(λ) and one for the exploration/exploitation algorithm].

A comparison of the exploration/exploitation model with Sarsa(λ) using an ε-greedy action selection rule is presented in Figure 2 for the learning environment used in Experiment 1.

All models details and fitting procedures are provided in the Appendix in Supplementary Material.

## Experiments

### Experiment 1: 2D-embeddable vs. non-embeddable environments

Our first experiment was designed to test whether there are performance differences between 2D-embeddable (Figure 1C) and 2D-non-embeddable environments (Figure 1B). We reasoned that an embeddable structure might facilitate the building-up of an internal, explicit representation, i.e., a map of the environment. If participants take advantage of this structure, then a model-based reinforcement learning algorithm like Dyna-Q should provide the best fit to the data.

### Stimuli and procedure

We used 11 images (plus the image “Yeah!” associated with the goal-state) and we varied whether or not the environment structure was embeddable. Participants were instructed (via written instructions) to find the goal-state as often as possible within 15 min. The assignment of images to state numbers was randomized from subject to subject such that all subjects had the same state-action transition matrix within each condition, but each subject had different images representing the different states. The starting states for each episode were limited to the four images farthest away from the goal-state and were selected randomly on each new episode. The position of the goal state was fixed at the beginning of each condition through the state-action transition matrix and did not change across episodes, i.e., it was independent of the starting states. For each of the two structure conditions, a new image set was employed.

In addition, participants were asked to “draw how to get from one image to another, using circles and arrows” via pen and paper at the end of each condition. Six participants participated in this experiment. The order of the conditions was randomized across participants.

## Results

### Experiment 1A

**Figure 3:**
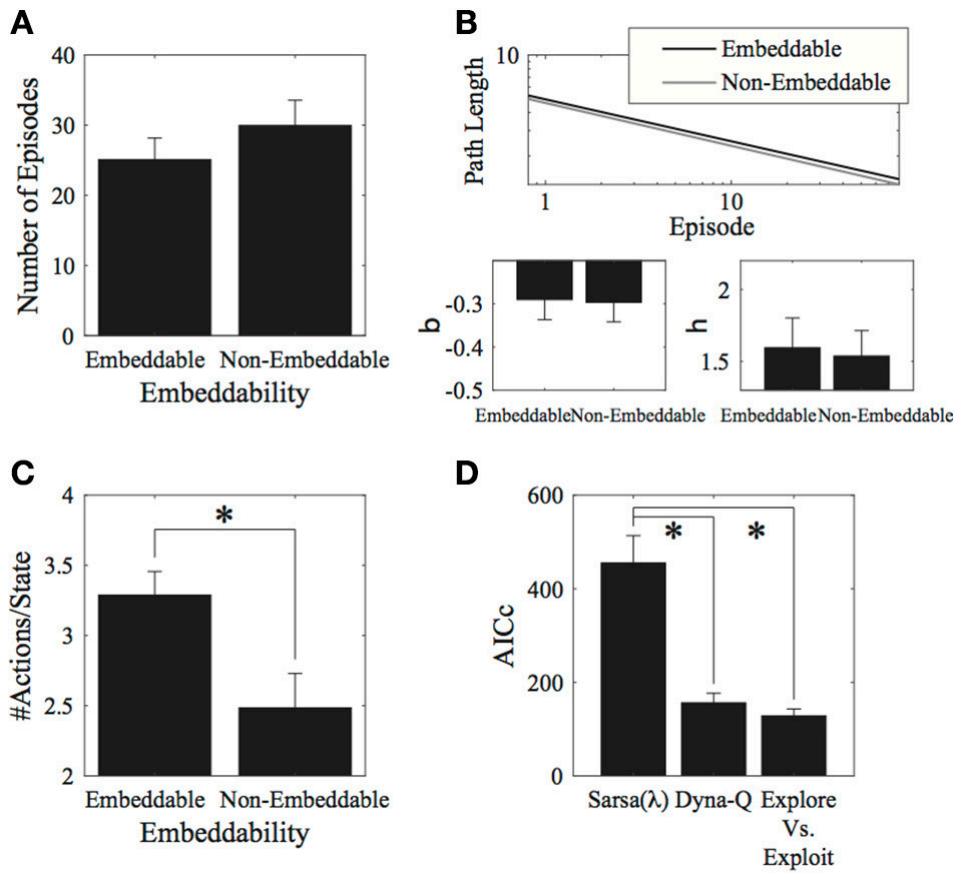
**Results for the environment structure. (A)** Number of episodes for the two conditions did not significantly differ. **(B)** Parameter fits for the two conditions also did not significantly differ. **(C)** Number of actions visited per state. Participants explored significantly more in embeddable than in non-embeddable environments. Error bars denote ±1 SEM for six participants. **(D)** Model comparisons (lower AICc values imply greater support for the given model). The exploration vs. exploitation model provides the best account of the data. ^*^p < 0.05 for the indicated comparisons.

Contrary to our expectations, we found little effects of the environment's 2D-embeddability on performance. With measure I, there was no significant effect of embeddability on the number of episodes completed [*t*_(5)_ = 1.07, *p* = 0.334, 2-tailed; Figure 3A]. No effect of embeddability was found even when considering the entire data set, without cutting off trials at the group minimum. Similarly, for measure II, there was no effect of embeddability on the log-log fit parameters [β: *t*_(5)_ = −0.09, *p* = 0.928, 2-tailed; η: *t*_(5)_ = −0.21, *p* = 0.843, 2-tailed; Figure 3B].

Interestingly, the embeddable environment leads to more exploration [*t*_(5)_ = −2.84, *p* = 0.036, 2-tailed, Cohen's *d* = 1.158—large effect; Figure 3C]. However, this does not lead, as mentioned, to superior performance for either the number of episodes (measure I), or the path length (measure II). Thus, subjects do not retain the extra information they gain from exploring more in the embeddable condition to find shorter paths to the goal. This result is surprising since a higher exploration rate in a more easily remembered condition should lead to better performance, particularly, when map formation is involved, however, this seems not to be the case, suggesting that subjects forget some of the states they explore.

**Figure 4:**
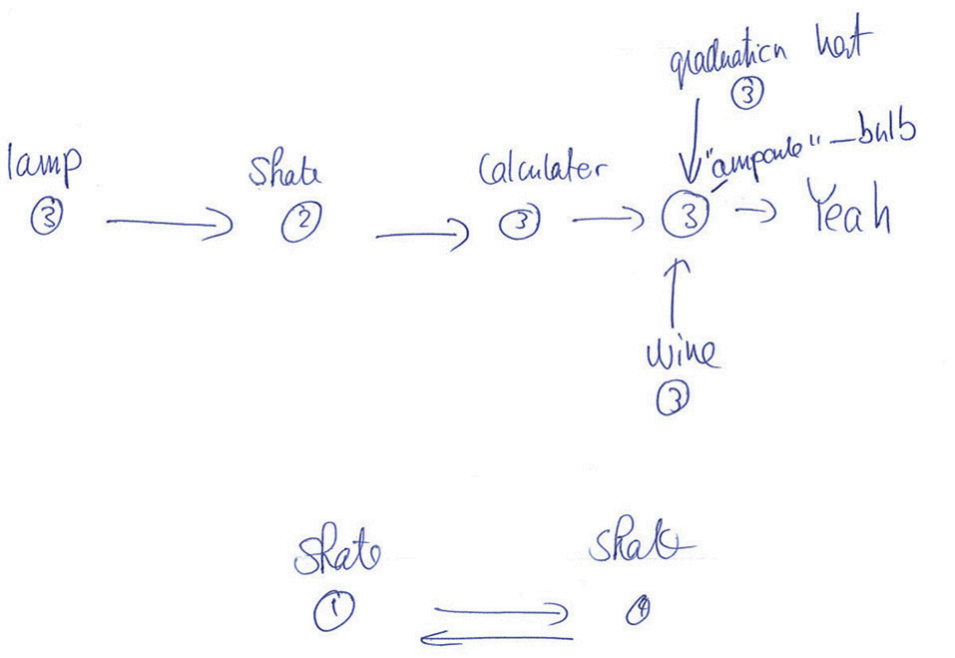
**Example drawing for one observer for the structure experiment (non-embeddable condition)**. In this typical example, the observer remembers some paths to the goal, but not the full structure of the environment (compare with Figure 1B).

This conclusion is also supported by the participants' drawings. After the experiment proper, participants were asked to reconstruct as much of the state-action decision space as they could, using a pen and paper. Examination of the sketches indicated that participants primarily remembered isolated chunks of the environments rather than remembering its full structure, even in the easy embeddable condition (see Figure 4 for a typical example). These results are further indication that the environment's structure has little effect. It may well be that participants perform the task with minimal knowledge about the environment and make their decisions based solely on the *Q*-values at each state, i.e., without making predictions beyond the current and subsequent state. Future research needs to address this question.

Next, we fitted model parameters to the data for the Sarsa(λ), Dyna-Q, and our Exploration/Exploitation model. The Exploration/Exploitation model best captured the participants' data (Figure 3D). This result holds true for each individual experimental condition. For the parameter fits to the data, no significant differences were found between structure conditions for any of the models (see Appendix A.4 in Supplementary Material, Tables 1–3 for the parameter values and Table 4 for comparative statistics).

For this experiment we also tried to fit the data to the Successor representation (SR) model (Dayan, 1993; Gershman et al., 2012; Momennejad et al., 2016) and to a hybrid mixture (HM) of model-free and model- based algorithms (Gläscher et al., 2010), to check whether our participants' learning strategy rather relies on a combination of the two algorithms.

The SR model lies in between pure model-free and pure model-based RL model; it exploits the knowledge that states can have similar successors by encoding the expected future visitations of each state along given trajectories (Appendix A.7 in Supplementary Material). The results of the SR model fits revealed significant differences between conditions in the learning rate (α) and eligibility trace (λ), but not in the exploration rate (τ). For both significant cases, the non-embeddable condition had higher parameter values than the embeddable condition (see Table 17 in Supplementary Material). The corresponding AICc values for this model, however, were somewhere between those for Dyna-Q and Sarsa(λ), indicating that this model does not explain the data as well as the Explore/Exploit model, but does a better job than Sarsa(λ) (Table 18 in Supplementary Material). Thus the results from this model's parameter fits remains somewhat equivocal.

We also fit a model that takes a weighted average between Sarsa(λ) and Dyna-Q in making action selections (i.e., a hybrid model). Here, the model has one extra parameter (*w*) relative to Sarsa(λ), which weights between Sarsa(λ) and Dyna-Q decisions (Appendix A.8 in Supplementary Material). Model fits for this model yielded no significant differences as a function of embeddability condition (see Table 19 in Supplementary Material). Furthermore, the corresponding AICc values for this model were much worse than Sarsa(λ) because of the extra parameter. We thus, exclude this model in the remaining experiments.

### Experiment 1B

To ensure that the null result in Experiment 1a was not due to insufficient training, we replicated Experiment 1a with a sample of 9 participants, who underwent a training session of 15 min in a different, non-embeddable environment, before the experiment proper. The procedure was otherwise identical to the procedure of Experiment 1a.

Results revealed no effect of embeddability on the number of episodes completed [*t*_(8)_ = −0.22, *p* = 0.830, 2-tailed], no effect of embeddability on the slopes of the log-log fit parameters [β: *t*_(8)_ = 0.41, *p* = 0.694, 2-tailed; η: *t*_(8)_ = 0.90, *p* = 0.396, 2-tailed], no effect of embeddability on the number of actions visited per state [*t*_(8)_ = −0.33, *p* = 0.753, 2-tailed]. Again, however, there was a significant effect of model type on AICc [*F*_(2, 16)_ = 24.21, *p* = 1.4 × 10^−5^], such that the Dyna-Q and the Explore/Exploit models had significantly better AICc values than the Sarsa(λ) model (both *p* < 0.05), but the Dyna-Q and Explore/Exploit models did not significantly differ from each other (*p* > 0.05). All other comparisons failed to reach statistical significance.

We found no effect of embeddability on any of our behavioral measures when pooling together data from Experiments 1a and 1b.

### Experiment 2A: ISI

Many reinforcement learning models keep track of the states that the agent visited in the past via the *eligibility trace*, whose length is controlled by the parameter λ. When λ = 0, the value of a given state is updated only when the agent visits it, i.e., there is no memory of previously visited states; when 0 < λ < 1 the value of a given state is updated—even if only to a minor extent—if the agent has visited it in the past. The state's trace gradually decays with time since it was last visited. Eventually, if the state has not been visited for a while, its value will not be updated. When λ = 1, the model has perfect memory: at each time step all previously visited states are updated and their traces do not decay with time (Sutton and Barto, 1998).

One unknown in this process is whether the eligibility trace decays with each trial or with absolute time, for example, related to a decay of dopamine levels or other molecular mechanisms (Fosnaugh et al., 1995; Plath et al., 2006). In models, the two are confounded. To investigate this question, we varied the ISI between image states in Experiment 2. We reasoned that if a long ISI negatively affected performance this would provide an indication that the eligibility trace decays with absolute time. If there were no effect on performance, then the eligibility trace would rather decay with the number of states.

### Stimuli and procedure

The environment consisted of 11 different images. The structure of the environment was *non-embeddable* (as in Figure 1B). Three out of ten images led directly to the goal-state. The initial image (at the beginning of each episode) was randomly chosen among all states which did not lead directly to the goal-state. We measured performance in three different conditions in which we provided ISI's of 0.5, 2, and 8 s. Different image sets were used in the three ISI conditions. Each ISI condition had a different total duration in order to have, on average, the same number of episodes in each of them. The duration of each condition was computed from the previous experiment and from a pilot. The long ISI condition lasted 40 min; the medium ISI condition lasted 12 min; the short ISI condition lasted 8 min, so that participants could complete an average of 35 episodes per condition (as estimated through pilot experiments). Each condition was run just once. Since the long ISI condition was very long, we divided it into two runs of 20 min each, one after the other with a break in between. We counterbalanced the order of the three ISI conditions across participants. Participants were instructed (via written instructions) to reach the goal state as often as possible. Eleven participants participated in the experiment.

### Results

**Figure 5:**
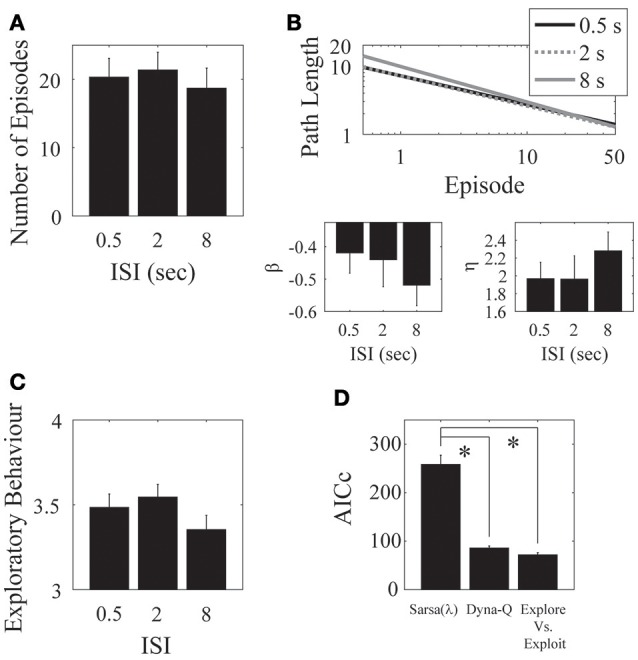
**ISI. (A)** There was no clear difference in the mean number of episodes completed in the 0.5, 2, and 8 s ISI conditions. **(B)** Parameter fits show a similar outcome of no effect over the three ISIs. **(C)** The mean number of actions visited per state is also not significantly different for the three ISIs. Error bars denote ±1 SEM for 11 participants. **(D)** Model comparison for the ISI experiment. Here, the Dyna-Q and “exploration vs. exploitation” models provide the best account of the data. ^*^p < 0.05 for the indicated comparisons.

We found no significant effect of ISI on the number of episodes completed [*F*_(2, 30)_ = 0.33, *p* = 0.722; Figure 5A], on the asymptotic performance, or on the learning rate [β: *F*_(2, 30)_ = 0.64, *p* = 0.535; η: *F*_(2, 30)_ = 0.33, *p* = 0.722; Figure 5B]. There was also no significant effect of ISI on exploratory behavior [*F*_(2, 30)_ = 0.48, *p* = 0.6245; Figure 5C].

Hence, our results support the notion that the eligibility trace decays with the number of visited states rather than with absolute time. One interpretation is that, for example, when playing chess what matters is the number of moves and not so much how much time it took to make them.

We found a significant effect of ISI on the λ parameter of the Sarsa(λ) model, such that the average of the short and medium ISI conditions were significantly different from the long ISI condition [*F*_(1, 20)_ = 6.7222, *p* = 0.01740]. We found no other effects of ISI on any of the remaining Sarsa(λ), Dyna-Q, or the Exploration/Exploitation parameter fits (Tables A.7, A.8, A.9, and A.10 in Supplementary Material), indicating that the same parameter setting in each model well-described subject performance in each condition. We again found a significant effect of Model on AICc [*F*_(2, 20)_ = 71.98, *p* = 7.29 × 10^−10^] such that that the Dyna-Q and “exploration vs. exploitation” models best captured the whole data set for this experiment [Sarsa(λ) vs. Dyna-Q: *F*_(2, 20)_ = 49.53, *p* = 1.79 × 10^−8^; Sarsa(λ) vs. Explore/Exploit: *F*_(2, 20)_ = 58.10, *p* = 4.66 × 10^−9^; Dyna-Q vs. Explore/Exploit: *F*_(2, 20)_ = 0.34, *p* = 0.715; Figure 5D]. This result holds for all experimental conditions.

As a final analysis, we split the episodes into first and last quarters and re-fit the model parameters within each quarter to see if there were any changes from the beginning to the end of the experiment (see Appendix A.6 in Supplementary Material for detailed parameter fits). Results revealed no significant main effects or interactions with ISI for the λ or τ parameters of the Sarsa(λ) model (all *p* > 0.05). There were significant main effects of ISI and Quarter on α [ISI: *F*_(2, 38)_ = 9.31, *p* = 5.11 × 10^−4^, Quarter: *F*_(1, 19)_ = 8.40, *p* = 0.009], but no significant interaction [*F*_(2, 38)_ = 0.44, *p* = 0.646]. α-values were higher for the 2 s ISI than for the 0.5 or 8 s ISI (both *p* < 0.05), but the 0.5 s ISI was not significantly different from the 8 s ISI (*p* > 0.05), and the results from the last quarter had higher α-values than the first quarter at all ISI's (all *p* < 0.05). This finding was not replicated in the Dyna-Q model, where no significant main effects or interactions of Quarter or ISI were found for the α-parameter (all *p* > 0.05). For the τ-parameter, however, there was a significant main effect of quarter [*F*_(1, 19)_ = 12.25, *p* = 0.002] such that the last quarter had lower τ-values than the first quarter. There was no main effect of ISI [*F*_(2, 38)_ = 0.3, *p* = 0.74], and no ISI × Quarter interactions [*F*_(2, 38)_ = 0.27, *p* = 0.77]. This suggests that exploration rates decrease as learning progresses. This sentiment was echoed by the Exploration/Exploitation model parameter fits, where for the *p*_*Explore*_ parameter, there was a significant main effect of Quarter [*F*_(1, 19)_ = 25.27, *p* = 7.49 × 10^−5^], but no main effect of ISI and no ISI × Quarter interaction (both *p* > 0.05). The parameter values for the first quarter were higher than for the last quarter (*p* < 0.05).

The effects of time on the α-, τ-, and *p*_*Explore*_-parameters suggest that as time passes, people learn faster paths to the goal and explore less.

## Experiment 2B: ISI

In Experiment 2a, we needed to vary the allotted time proportionally to the ISIs in order to properly compare the number of episodes completed. Here, we avoided this procedure by using a fixed number of episodes with no time pressure. We determined the total number of trials over all episodes. Good learners completed the task using fewer trials than poor learners.

### Stimuli and procedure

Twelve new subjects participated in this experiment and were each given as much time as they needed to complete 50 episodes. We used ISI's of 0.5, 2, and 6 s. Otherwise the conditions were identical to Experiment 2a.

### Results

**Figure 6:**
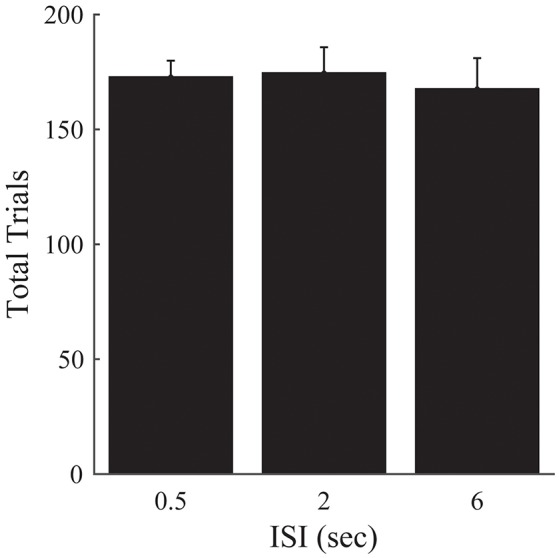
**Experiment 2B**. Total trials completed over all episodes as a function of ISI. Error bars represent ±1 SEM. There are no significant differences between the different ISI conditions. Since the other analyses presented in Figure 5 are robust to differences in the amount of time provided to perform the task, we omit them here.

There was no significant effect of ISI on the number of trials required to complete the 50 episodes [*F*_(2, 33)_ = 0.15, *p* = 0.859; Figure 6].

These results corroborate our previous findings, and suggest that the previous lack of an effect of ISI on the number of episodes completed is not attributable solely to differences in the time provided to complete the task for a given ISI condition.

## Experiment 3: monetary incentive

Given the crucial nature of reward in reinforcement learning models, we investigated whether monetary incentives influence learning. We tested two groups of participants. We kept the total monetary reward fixed for both groups while manipulating whether or not reward delivery was performance-contingent between the groups—one group was paid based on their performance while, in the other group, participants' payments were matched to those of the participants in the performance-contingent reward group.

### Stimuli and procedure

The environment consisted of 11 different images and was non-embeddable (Figure 1B). The goal-state was reachable from four states. The initial images were randomly selected from among those farthest away from the goal-state. The first group of participants (*n* = 15; the “flexible reward” group) was instructed (via written instructions) to reach the goal-state as often as possible. They were informed that each time they reached the goal-state, they would receive 0.25 Swiss Francs. The second group of participants (*n* = 15; the “fixed reward” group) performed the same experiment but received a fixed reward at the end of the experiment, independent of how many times they reached the goal-state. The amount of reward provided to each individual in the *fixed reward* group was matched to the reward provided to a corresponding individual from the first group.

### Results

**Figure 7:**
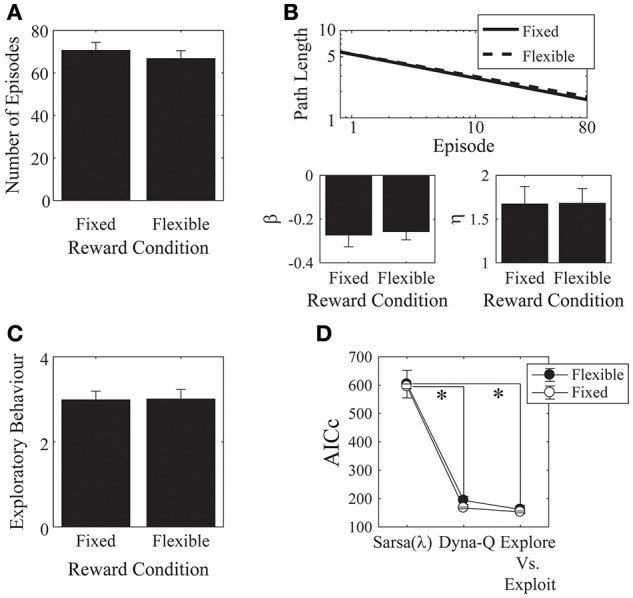
**Monetary reward**. **(A)** There was no difference in the mean number of episodes completed between the fixed- and the flexible-reward groups. **(B)** Parameter fits for each of the reward conditions showed no significant difference between the two groups. **(C)** The mean number of actions visited per state was not significantly different between the fixed and flexible groups. Error bars denote ±1 SEM for 15 participants. **(D)** Models comparison: here, the Dyna-Q and the “Exploration vs. Exploitation” models provide significantly better accounts of the data than the Sarsa(λ) model. ^*^p < 0.05 for the indicated comparisons.

We found no effects of the monetary incentive on *any* of our measures, neither on the number of episodes [*t*_(28)_ = 0.73, *p* = 0.471, Cohen's *d* = 0.27; Figure 7A], nor on asymptotic performance, nor on the initial learning rate [β: *t*_(28)_ = −0.23, *p* = 0.817, Cohen's *d* = 0.09, 2-tailed; η: *t*_(28)_ = −0.03, *p* = 0.976, Cohen's *d* = 0.01, 2-tailed. Figure 7B], nor on the exploratory behavior [Figure 7C: *t*_(28)_ = −0.06, *p* = 0.952, Cohen's *d* = 0.02, 2-tailed]. We also checked out the “number of episodes completed” measure to see if any effects emerged when we included all trials, instead of cutting off each subject's data at the group minimum. This analysis also revealed no effect of monetary incentive.

We found significant effects of incentive on the α and λ parameters of the Sarsa(λ) model [α: *t*_(28)_ = 2.36, *p* = 0.03, Cohen's *d* = 0.86, λ: *t*_(28)_ = −2.03, *p* = 0.05, Cohen's *d* = 0.74]. α was higher in the flexible-reward condition, while λ was higher in the fixed reward condition. Otherwise, there were no significant effects of incentive on any of the remaining parameters for any of the models (all *p* > 0.05; Appendix A.4 in Supplementary Material: parameters are provided in Tables 11–13, statistical comparisons are provided in Tables 14–16). We found that, in both groups, the Dyna-Q and Exploration vs. Exploitation models provided significantly better accounts of the data than the Sarsa(λ) model [Figure 7D; Model: *F*_(2, 56)_ = 176.18, *p* = 6.92 × 10^−25^, partial-eta^2^ = 0.863; Incentive: *F*_(1, 28)_ = 0.497, *p* = 0.495, partial-eta^2^ = 0.02; Model × Incentive: *F*_(2, 56)_ = 0.081, *p* = 0.922, partial-eta^2^ = 0.003; Sarsa(λ) vs. Dyna-Q: *p* = 2.95 × 10^−14^; Sarsa(λ) vs. Explore/Exploit: *p* = 3.32 × 10^−14^; Dyna-Q vs. Explore/Exploit: *p* = 0.144].

## Discussion

Classic RL models are distinguished by (1) whether or not they build up a map of the environment (i.e., “model-based” vs. “model-free” learning), (2) the use (or not) of an eligibility trace, and (3) the primacy of external rewards. We found very little evidence that these elements play a pivotal role for human learning.

First, human participants do not seem to build up a complete map of the environment, as indicated by the line drawings showing that participants only remember short path segments leading to the goal and not full maps (Figure 4). Furthermore, participants seem to be relatively unaffected by whether or not the state-action connectivity matrix was 2D-embeddable. This holds true at least for the learning periods we used. More extensive training may potentially lead to more elaborate map formation, however we did not observe exhaustive map formation even with participants who completed as many as 82 episodes. Moreover, we did not find any effect of the 2D-embeddable structure in our control experiment (Experiment 1b) in which a different group of participants (*n* = 9) underwent a longer training session.

In embeddable environments, it is possible to infer the optimal action even for states that were never visited. For example, in Figure 1C one can infer that taking action 2 from state 6 will lead to state 7, where one should subsequently take action 2 to get to the goal state. Our findings, however, suggest that humans do not seem to rely on this information to find the shortest path to the reward.

Second, we found that learning was unaffected by ISI, lending support to the hypothesis that the eligibility trace is updated as a function of the number of trials, or learning events, and not as a function of a fixed time interval. This suggests that the human reinforcement learning system is not limited by a single fixed-duration neurotransmitter-based processes, such as transient dopamine concentrations, and instead, is robust enough to integrate information over multiple time scales.

Third, external reward seems to play only a minor role since we found no difference in learning performance regardless of whether or not participants received performance-based monetary compensation. However, it might well be that humans receive internal rewards for learning task-relevant skills that are independent of the monetary rewards they receive. This would explain, for example, why people play video games that provide no monetary compensation. It is worth mentioning that participants enjoyed our paradigm and, possibly, found reaching the goal rewarding enough to outweigh the added incentive of making money. However, other studies, using different experimental paradigms, have reported the opposite result. For example, it has been shown that when offered monetary compensation, participants performed better in a delayed memory task, which has led to the hypothesis that monetary incentives promote memory formations via hippocampal dopamine release (Adcock et al., 2006).

### Model results and implications

In most experiments, we found no significant behavioral effects and concomitantly no significant parameter difference in our model fits.

For example, with an embeddable environment structure, one might expect that subjects are able to exploit regular structure to retain more information about the environment. In the model, any additional retained information about past states manifests as an increase in the λ parameter of the Sarsa(λ) algorithm. Our model fits revealed that this parameter was not significantly higher for the embeddable condition than for the non-embeddable condition of Experiment 1. Likewise, participants' performance in the two conditions was comparable.

However, we found significantly different parameter fits when comparing short vs. long ISIs and fixed vs. flexible reward conditions. These differences may be explained by the fine-grained nature of the model parameter fits, which take into account trial-by-trial variability. Our behavioral measures, like the number of completed episodes, are coarser in scale, and pool together information from all trials, thereby losing some of the details about how learning progressed throughout the experiment. The model parameter fits reflect underlying features of how subjects learn and the differences suggest that the eligibility trace (parameterized by λ in our models) varies as a function of ISI, taking into account the most information for short and medium ISI's and falling off with higher ISI's. For the reward experiment, the higher α and lower λ parameters for the flexible condition indicate that subjects integrate information over fewer past states, but weight this information more heavily than in the fixed reward condition—possibly indicating an urgency to find a good solution as fast as possible so as to maximize gains within the 15 min allotted for the experiment.

In terms of which model best describes human learning for our paradigm, we found that the exploration/exploitation model had the best (i.e., lowest) Akaike Information Criterion scores in all experiments. This is likely due to our use of a task with deterministic state-action transitions. Here, participants can simply memorize which actions lead to which states. With stochastic state-action transitions, however, the probability of transferring to each state must be learned for each state-action pair. Classic reinforcement learning models such as Sarsa(λ) and Dyna-Q integrate information over many trials to estimate the value of each state and action or the probabilities of the state-action transitions within the environment. Our Exploration/Exploitation algorithm, on the other hand, knows a-priori that our state-action transitions are deterministic and needs only to memorize which actions lead to which states. This type of learning is much faster (Figure 2) and, interestingly, humans seem to be able to capitalize on this fact, thereby matching the algorithm's performance on all of our tasks.

### Relation to other experimental paradigms

There are a plethora of one-stage decision making experiments where choosing between two or more options entails an immediate outcome (Hanes and Schall, 1996; Shadlen and Newsome, 1996, 2001; Schall and Thompson, 1999; Gold and Shadlen, 2000; Sugrue et al., 2004; Daw et al., 2006; Dayan and Daw, 2008; Wittmann et al., 2008; Ito and Doya, 2009; Krajbich et al., 2010). Even though these experiments can capture basic decision-making situations, it remains an open question whether sequential decision making, which requires multiple decision stages to attain the final outcome, can be modeled as a linear concatenation of such basic processes. Very recent research has addressed this issue using a two-stage decision making paradigm, in which participants had to make two binary choices before receiving feedback about rewards (Solway and Botvinick, 2015). There the authors show that standard decision-making models, in which evidence about task-relevant variables is accrued in time until a choice is made, can be extended at least to two-stage decision-making problems. The same binary decision tree, but with stochastic state transitions, has been used by Gläscher et al. (2010) to examine behavioral learning performance and simultaneously track correlated BOLD activations. Our paradigm, has been leveraged to probe learning effects over a much larger and more complex state space, in which, contrary to the previous studies, paths in the decision trees are not necessarily disjointed, i.e., the same state may be visited through more than one path to the goal. These features not only bring our paradigm closer to real-life situations, but also allow examining the effects of the state space's layout on learning performance (e.g., in our embeddable vs. non-embeddable experiments). Furthermore, we showed how our paradigm could flexibly be adapted to answer questions about stimulus timing, and reward.

### Outlook

Here we have shown the versatility and flexibility of our new behavioral paradigm for testing human reinforcement learning. Several interesting questions have been answered, such as the influence of 2D-embeddability on learning, the effect of stimulus timing on learning, and the influence of monetary rewards on learning. We believe that these results are just a starting point for the systematic investigation of sequential decision-making beyond two states. For example, it would be interesting to investigate which environments lead to faster learning than others based on different graph theoretical measures. Studies like this may be of crucial interest for how to organize decision making in human navigation, sports, medical surgery, or work processes, where several actions need to be executed in order to complete a task.

## Author contributions

ET and MH designed the study. ET and AC analyzed the data and performed the simulations. The three authors wrote the paper.

### Conflict of interest statement

The authors declare that the research was conducted in the absence of any commercial or financial relationships that could be construed as a potential conflict of interest.
