# Supplementary material for: What to Choose Next? A Paradigm for Testing Human Sequential Decision Making
Source: Front Psychol. 2017 Mar 7;8:312. doi: 10.3389/fpsyg.2017.00312 (PMC5339299; doi:10.3389/fpsyg.2017.00312)
Supplement: Supplementary file 1 [file DataSheet1.docx]

# Appendix A.

## Modeling

### Sarsa(λ)

We fitted the data to the standard Sarsa(λ) reinforcement learning model (Sutton and Barto, 1998). We used softmax action selection and the eligibility-trace (with replacement-trace) procedure (Sutton & Barto, 1998). Pseudo-code for the algorithm with our maximum likelihood procedure (described next) is provided in Appendix A.1. We first calculated the likelihood of selecting each of the four possible actions on each episode using the softmax action probability:

$p\left( a_{i},s_{i} \right)=\frac{e^{\frac{Q\left( a_{i},s_{i} \right)}{\tau}}}{\sum_{j=1}^{N_{a}} e^{\frac{Q\left( a_{j},s_{i} \right)}{\tau}}}$ (A. 1)

where *a_i_* denotes the action under consideration at the current state *s_i_,* *N_a_* is the total number of possible actions at this state (four in our experiments), *Q*(*a_i_,* *s_i_*) is the action-value function for action *a_i_* at state *s_i_* (which the agent learns over trials), τ controls the degree of exploration the model indulges in (higher values mean more exploration) and *p*(*a_i_*, *s_i_*) specifies the probability that the model will choose action *a_i_* on the current trial given that the learning agent is in state *s_i_*. The log-likelihood of the observed data, under a given set of model parameters is then:

$L\left( \alpha,\lambda, \tau\right)= \sum_{i}^{N_{t}} \left[ ln\left( p\left( a_{i},s_{i} \right) \right)+\sum_{j\neq i} ln\left( 1-p\left( a_{j},s_{i} \right) \right) \right]$ (A. 1)

Where α controls the learning rate, λ the decay of the eligibility trace, *a_i_* is the action the participant chose on trial *i* and *N_t_* is the total number of trials, pooling over all episodes. Maximizing the data’s likelihood given the model parameters yielded estimates for α, λ, and τ for each observer. We then compared the values of the model’s parameters averaged across participants between the different experimental conditions using a repeated measures ANOVA (or t-test) for each experiment.

### Dyna-Q

Next, we fitted individual observer’s data with a model-based reinforcement learning algorithm, which, contrary to Sarsa(λ), incorporates knowledge of the environment structure. We implemented a Dyna-Q learning algorithm with free parameters α and τ (defined as in Sarsa(λ)) and with a fixed simulation time of 10 state-action selections (Sutton and Barto, 1998). Fitting proceeded, as for the Sarsa(λ) algorithm, by finding the model parameters that maximized the log-likelihood of the data using equations A.1 and A.2. Pseudo-code for the algorithm and maximum likelihood procedure is provided in Appendix A.2.

### Exploitation Versus Exploration

We developed a simplified single-parameter algorithm (Fig.1), which memorizes shortest paths to the goal (which might be a strategy used by the participants given the rather small size of our environments). The model makes a choice at each state of whether to explore or exploit. Here, exploring means trying previously untried actions and exploiting means taking previously tried actions that are known to lead to the goal. The model determines whether to explore or exploit probabilistically using the free parameter *P*(*Explore*). When exploring, the model chooses randomly between the remaining unexplored actions. When the goal is reached, the state immediately leading up to the goal is added to a look-up table indicating its distance to the goal. The second time the goal is reached from this same state, the penultimate state is added to the look-up table. In this way a memory trace is implemented that updates the list of remembered states by one for each episode. When exploiting, these table entries are compared for all known states, and the state with the shortest distance to the goal is selected. If all actions have been tried then *P*(*Explore*) is set to zero (since no unexplored states remain) and the model takes the fastest known path to the goal. The update equations are simply look-up table entries such as: D(s_2_; a_4_) ← 3, where D is the distance to the goal, and (s_2_; a_4_) denotes state 2, action 4. This model is illustrated schematically in Appendix A.3 and is followed by Matlab code for its implementation.

### Akaike Information Criterion

Results from each model are compared using the Akaike Information Criterion adjusted for finite sample sizes (AICc; Appendix A.5). This measure provides a common metric for model comparison that takes into account both the fit residuals and the numbers of parameters in each model. In other words, AIC penalizes models with more free parameters. Lower AICc values indicate a better model, i.e. a better fit to the data.

## Appendix A.1. Sarsa(λ) learning algorithm

Initialize *Q*(*s*; *a*) arbitrarily and *e*(*s*; *a*)= 0, for all *s*, *a*

For each episode:

Initialize *s_old_*, *a_old_*

While *s_old_* is not a goal state:

Take action *a_old_*, observe reward *r* and state *s_new_*

For each possible action a_i_:

$$q\left( i \right)\leftarrow\frac{exp\left( \frac{Q\left( s_{new},a_{i} \right)}{\tau} \right)}{\sum_{j} exp\left( \frac{Q\left( s_{new},a_{j} \right)}{\tau} \right)}$$

End for loop.

Choose *a_new_* from the subject’s actions (normally the algorithm would choose *a_new_* by randomly sampling from the distribution defined by *q*)

The likelihood of *a_new_* is given by:

$L=ln q\left( a_{\mathrm{chosen}} \right)+\sum\ln\left( 1-q\left( a_{not chosen} \right) \right)$

Update parameters:

$\delta\leftarrow r+Q\left( s_{\mathrm{new}},a_{\mathrm{new}} \right)-Q\left( s_{\mathrm{old}},a_{\mathrm{old}} \right)$

$e\left( s_{\mathrm{old}},\left[ a\notin a_{\mathrm{old}} \right] \right)\leftarrow0$

$e\left( s_{\mathrm{old}},a_{\mathrm{old}} \right)\leftarrow e\left( s_{\mathrm{old}},a_{\mathrm{old}} \right)+1$

For all *s*, *a*:

$Q\left( s,a \right)\leftarrow Q\left( s,a \right)+\alpha\cdot\delta\cdot e\left( s,a \right)$

$e\left( s,a \right)\leftarrow\lambda\cdot e\left( s,a \right)$

End for loop.

$s_{\mathrm{old}}\leftarrow s_{\mathrm{new}}$

$a_{\mathrm{old}}\leftarrow a_{\mathrm{new}}$

End while loop.

End for loop.

## Appendix A.2. Dyna-Q learning algorithm

Initialize *Q*(*s*; *a*) arbitrarily and *e*(*s*; *a*) = 0, *M*(*s*,*a*,*s_new_*,*r*) = 0 for all *s*, *a*, *s_new_* and *r*

For each episode:

Initialize *s_old_*, *a_old_*

While *s_old_* is not a goal state:

Take action *a_old_*, observe reward *r* and state *s_new_*

For each possible action a_i_:

$$q\left( i \right)\leftarrow\frac{exp\left( \frac{Q\left( s_{new},a_{i} \right)}{\tau} \right)}{\sum_{j} exp\left( \frac{Q\left( s_{new},a_{j} \right)}{\tau} \right)}$$

End for loop.

Choose *a_new_* from the subject’s actions (normally the algorithm would choose *a_new_* by randomly sampling from the distribution defined by *q*).

The likelihood of *a_new_* is given by:

$L=ln q\left( a_{\mathrm{chosen}} \right)+\sum\ln\left( 1-q\left( a_{not chosen} \right) \right)$

Update parameters:

$\delta\leftarrow r+Q\left( s_{\mathrm{new}},a_{\mathrm{new}} \right)-Q\left( s_{\mathrm{old}},a_{\mathrm{old}} \right)$

For all *s*, *a*:

$Q\left( s,a \right)\leftarrow Q\left( s,a \right)+\alpha\cdot\delta$

End for loop.

$M\left( s_{\mathrm{old}},a_{\mathrm{old}},1 \right)\leftarrow s_{\mathrm{New}}$

$M\left( s_{\mathrm{old}},a_{\mathrm{old}},2 \right)\leftarrow r$

For 10 iterations:

$s_{\mathrm{sim}}\leftarrow$ random previously observed state

$a_{\mathrm{sim}}\leftarrow$ random action previously taken in s


$$Q\left( s_{sim},a_{sim} \right)\leftarrow Q\left( s_{sim},a_{sim} \right)+\alpha\left[ r+\max_{a_{{sim}_{New}}} Q\left( s_{{sim}_{New}},a_{{sim}_{New}} \right)-Q\left( s_{sim},a_{sim} \right) \right]$$

End for loop.

$s_{\mathrm{old}}\leftarrow s_{\mathrm{new}}$

$a_{\mathrm{old}}\leftarrow a_{\mathrm{new}}$

End while loop.

End for loop.

## Appendix A.3. Exploration versus Exploitation


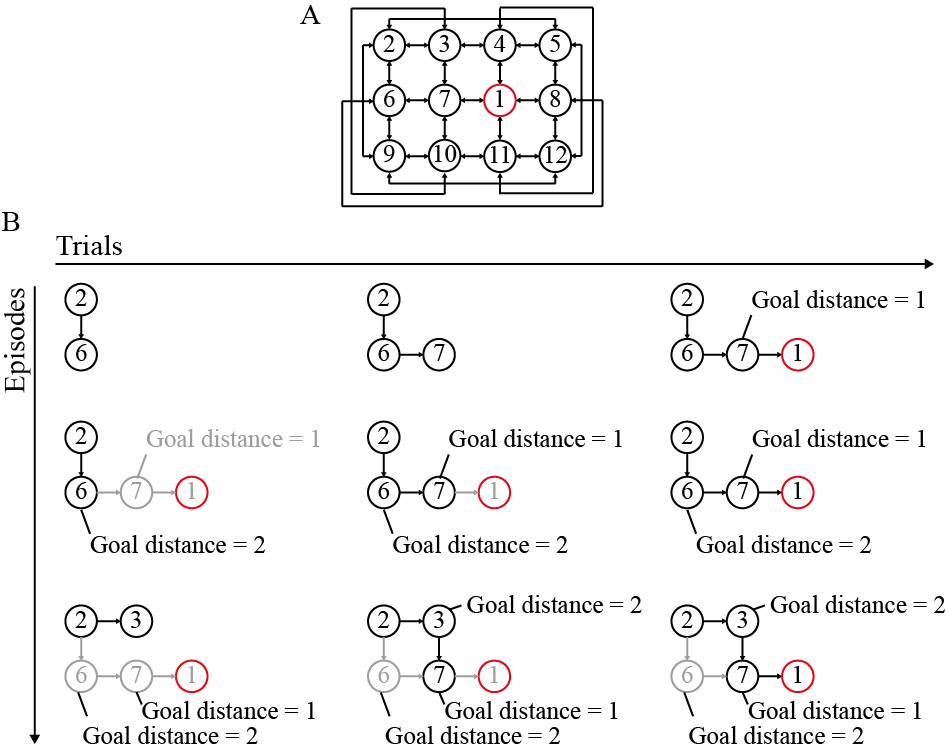


Figure 1. A. The state-action transition structure for the environment in the following example. States are nodes marked with circled numbers and actions are marked by the arrows connecting the different states. State 1 is the goal state (outlined in red). B. Demonstration of the exploration/exploitation learning algorithm. Going left to right shows the algorithm’s representation of the space along subsequent trials within an episode. Going top to bottom shows the representation along subsequent episodes. Initially, in the first episode, the algorithm explores randomly until the goal state is reached. Once the goal has been reached the penultimate state is updated with the distance to the goal. On subsequent episodes, when this penultimate state is visited, the distance to the goal for the immediately previous state is also updated - thereby implementing an eligibility trace. The algorithm generally follows one of two possible options at each state: either it exploits the shortest known path to the goal, or it explores previously un-tested paths that *may* lead to the goal in fewer steps. The propensity to follow one option or the other is determined by a single parameter *p*(*explore*) that determines the probability of exploring/exploiting at any given state (*p*(*exploit*) = 1 - *p*(*explore*)). In cases where no paths have been explored *p*(*explore*) = 1 and in cases where all paths have been explored, the path leading to the goal in the least number of steps is always taken.

### Matlab code for the Exploration Vs. Exploitation algorithm:

function EpiLen = PathLearner(TransM,StartS,pExplore)

% PATHLEARNER Learns paths through a state action transition matrix to

% a goal state. The inputs are the state-action transition matrix, and a

% list of starting states. The state-action transition matrix is

% organized as a [Number of States x Number of Actions] matrix, with the

% first row being the goal state and the remaining rows indicating, for

% each state-action combination, which state that state-action

% combination leads to. The starting states are a one dimensional

% array indicating the desired starting state for each episode.

%

% Usage: EpiLen = PathLearner(TransM,StartS,pExplore);

%

% e.g.

%

% % State-action transition matrix

% TransM = [1 1 1 1

% 5 6 5 1

% 4 7 2 3

% 7 4 6 5

% 7 3 8 7

% 7 5 7 6

% 8 2 7 7

% 8 4 4 2];

% StartS = [2 5 7 5 5 2 2 7 5 2 5 7 7 5];

% pExplore = 0.5;

%

% EpiLen = PathLearner(TransM,StartS,pExplore);

%

% figure;

% plot(1:length(StartS),EpiLen,'r-');

% xlabel('Episode','FontName','Times New Roman','FontSize',16);

% ylabel('Path Length','FontName','Times New Roman','FontSize',16);

%

% Created by Aaron Clarke, EPFL, LPSY, Herzog Lab, April 10, 2013

% Initialize constants

nEpi = length(StartS); % Number of episodes

[nS,nA] = size(TransM); % Number of states and actions

EpiLen = ones(nEpi,1); % Episode length counter

% Initialize the node struct

node = struct('ChildrenS',zeros(1,nA),'GoalDist',zeros(1,nA));

for iS = 2:nS

node(iS).GoalDist = (nS+1)*ones(1,nA);

end

for iEpi = 1:nEpi

% Initialize the starting state for the current episode

S = StartS(iEpi);

StillSearching = (S~=1);

while StillSearching

% Update the episode length counter

EpiLen(iEpi) = EpiLen(iEpi)+1;

% Choose an action

if any(node(S).GoalDist~=(nS+1))

if all(node(S).GoalDist~=(nS+1))

% All actions have been explored.

% Choose the action that leads to the goal in the fewest

% steps.

[~,A] = min(node(S).GoalDist);

else

% Not all actions have been explored.

% Explore with probability "pExplore."

if rand(1)<=pExplore

% Randomly pick one of the unexplored actions

UnExplored = find(node(S).GoalDist==(nS+1));

A = UnExplored(unidrnd(length(UnExplored),1,1));

else

% Take the fastest action known

[~,A] = min(node(S).GoalDist);

end

end

else

% Choose a random action

A = unidrnd(nA,1,1);

end

% Observe the new state

sNew = TransM(S,A);

% Update the node tree

node(S).ChildrenS(A) = sNew;

if sNew==1

% We have reached the goal

node(S).GoalDist(A) = 1;

StillSearching = false;

else

% We are still searching for the goal.

% Update the distance to the goal counter.

sInd = find(node(S).ChildrenS);

if ~isempty(sInd)

for iS = 1:length(sInd)

node(S).GoalDist(sInd(iS)) = min(node(S).GoalDist(sInd(iS)),...

1+min(node(node(S).ChildrenS(sInd(iS))).GoalDist));

end

end

end

S = sNew;

end

end

## Appendix A.4. Data fitting

Table 1. Sarsa(λ) parameter fits for the structure experiment.

| Parameter | Embeddable | Non-Embeddable |
| --- | --- | --- |
| α | 0.63 ± 0.13 | 0.55 ± 0.06 |
| λ | 0.90 ± 0.03 | 0.90 ± 0.00 |
| τ | 0.65 ± 0.12 | 0.30 ± 0.10 |

Table 2. Dyna-Q parameter fits for the structure experiment.

| Parameter | Embeddable | Non-Embeddable |
| --- | --- | --- |
| α | 0.01 ± 0.01 | 0.32 ±0.21 |
| τ | 1.00 ± 0.59 | 1.24 ± 0.69 |

Table 3. Exploitation/exploration parameter fits for the structure experiment. Values are means over subjects ± standard errors.

| Embeddable | Non-Embeddable |
| --- | --- |
| 0.23 ± 0.07 | 0.15 ± 0.07 |

Table 4. Model parameter comparisons between the embeddable and non-embeddable environments for the structure experiment.

| Model | Parameter | t-statistic | p-value (two-tailed) |
| --- | --- | --- | --- |
| Sarsa(λ) | α | t(5) = -0.97 | p = 0.376 |
|  | λ | t(5) = 0.54 | p = 0.611 |
|  | τ | t(5) = -0.74 | p = 0.493 |
| Dyna-Q | α | t(5) = 1.22 | p = 0.278 |
|  | τ | t(5) = 1.12 | p = 0.312 |
| Exploration/exploitation | p_explore_ | t(5) = -0.68 | p = 0.526 |

Table 5. Sarsa(λ) parameter fits averaged over subjects in the short, medium and long ISI conditions of the ISI Experiment. Error bars denote ± SEM for the eleven participants.

| Parameter | 0.5 s | 2 s | 8 s |
| --- | --- | --- | --- |
| α | 0.34 ± 0.08 | 0.32 ± 0.08 | 0.56 ± 0.09 |
| λ | 0.97 ± 0.01 | 0.98 ± 0.01 | 0.93 ± 0.02 |
| τ | 0.44 ± 0.09 | 0.50 ± 0.12 | 0.56 ± 0.08 |

Table 6. Averaged Dyna-Q parameter fits over subjects in the short, medium and long ISI conditions of the ISI Experiment. Error bars denote ±1 SEM for the eleven participants.

| Parameter | 0.5 s | 2 s | 8 s |
| --- | --- | --- | --- |
| α | 0.04 ± 0.01 | 0.11 ± 0.09 | 0.09 ± 0.07 |
| τ | 0.28 ± 0.09 | 0.18 ±0.06 | 0.21 ± 0.08 |

Table 7. Probability of exploration for each ISI.

| 0.5 s | 2 s | 8 s |
| --- | --- | --- |
| 0.26 ± 0.10 | 0.51 ± 0.13 | 0.53 ± 0.09 |

Table 8. ANOVA results on Sarsa(λ) parameter fits to the different conditions of the ISI experiment.

| α | λ | τ |
| --- | --- | --- |
| F(2,30) = 2.35, p = 0.112 | F(2,30) = 3.44, p = 0.045 | F(2,30) = 0.70, p = 0.504 |

Table 9. ANOVA results on Dyna-Q parameter fits to the different conditions of the ISI experiment.

| α | τ |
| --- | --- |
| F(2,30) = 0.33, p = 0.721 | F(2,30) = 1.29, p = 0.290 |

Table 10. ANOVA results on exploration/exploitation parameter fits to the different conditions of the ISI experiment.

| EE |
| --- |
| F(2,30) = 2.27, p = 0.121 |

Table 11. Averaged Sarsa(λ) parameter fits over subjects in the fixed and flexible reward conditions of the reward experiment. Errors are ±1 SEM.

| Parameter | Fixed | Flexible |
| --- | --- | --- |
| α | 0.43 ± 0.08 | 0.68 ± 0.07 |
| λ | 0.93 ± 0.02 | 0.89 ± 0.02 |
| τ | 0.33 ± 0.05 | 0.37 ± 0.07 |

Table 12. Averaged Dyna-Q parameter fits over subjects in the fixed and flexible conditions of the reward experiment. Errors are ±1 SEM.

| Parameter | Fixed | Flexible |
| --- | --- | --- |
| α | 0.09 ± 0.06 | 0.03 ± 0.01 |
| τ | 0.20 ± 0.06 | 0.20± 0.05 |

Table 13. Probability of exploration as a function of reward contingency (means plus or minus standard errors). We find no effect of reward contingency on the probability of exploration.

| Fixed | Flexible |
| --- | --- |
| 0.27 ± 0.08 | 0.34 ± 0.09 |

Table 14. T-test results on Sarsa(λ) parameter fits of Reward Experiment.

| α | λ | τ |
| --- | --- | --- |
| t(28) = 2.36, p = 0.03 | t(28) = -2.03, p = 0.05 | t(28) = 0.37, p = 0.71 |

Table 15. T-test results on Dyna-Q parameter fits of Reward Experiment.

| α | τ |
| --- | --- |
| t(28) = -0.95, p = 0.35 | t(28) = -0.04, p = 0.97 |

Table 16. T-test results on exploration/exploitation parameter fits of Reward Experiment.

| EE |
| --- |
| t(28) = 0.56, p = 0.58 |

Table 17. Successor representation parameter fits and t-tests for the Structure experiment

| Parameter | Embeddable | Non-Embeddable | Statistics |
| --- | --- | --- | --- |
| α | 0.80±0.04 | 1.00±0.00 | t(14) = 4.83, p = 0.0003 |
| λ | 0.35±0.07 | 0.65±0.08 | t(14) = 4.04, p = 0.001 |
| τ | 0.33±0.07 | 0.37±0.08 | t(14) = 0.47, p = 0.642 |

Table 18. Successor representation AICc values for the structure experiment

| Embeddable | Non-Embeddable |
| --- | --- |
| 240.62±44.44 | 577.37±78.77 |

Table 19. Hybrid model parameter fits and t-tests for the Structure experiment.

| Parameter | Embeddable | Non-Embeddable | Statistics (two-tailed) |
| --- | --- | --- | --- |
| α | 0.79±0.06 | 0.80±0.04 | t(14) = 0.08, p = 0.937 |
| λ | 0.87±0.02 | 0.81±0.05 | t(14) = -0.95, p = 0.358 |
| τ | 0.28±0.06 | 0.25±0.06 | t(14) = -0.35, p = 0.728 |
| w | 0.70±0.07 | 0.73±0.05 | t(14) = 0.26, p = 0.796 |

Table 20. Hybrid model AICc values for the Structure experiment

| Embeddable | Non-Embeddable |
| --- | --- |
| 414.32±41.25 | 510.71±68.96 |

## Appendix A.5: Akaike Information Criterion

The Akaike Information Criterion adjusted for finite sample sizes (AICc) is computed in two steps as:

$$AIC=n\cdot ln\left( \frac{RSS}{n} \right)+2k$$

$$AICc=AIC+\frac{2k\left( k+1 \right)}{n-k-1}.$$

Here, *n* is the sample size (i.e., the number of subjects), *k* is the number of free parameters in the model, and *RSS* is the residual sum of squares ($RSS=\sum_{i} \left( x_{i}-\hat{x}_{i} \right)^{2}$ where *x_i_* are the human data and $\hat{x}_{i}$ are the model predictions).

## Appendix A.6: Comparison of Model Fits Over the First and Last Quarters of Episodes

For each experiment and each model, we split the data for each subject into quarters (i.e. first 25% of episodes and last 25% of episodes), and fit model parameters separately to the first and last quarter of the data.

## Structure Experiment

Parameter values are provided in Table 17.

Table 21. Model parameter fits for the structure experiment. Errors represent ±SEM.

| **Sarsa(λ)** |  |  |  |
| --- | --- | --- | --- |
| Embeddable: |  |  |  |
| Quarter | α | λ | τ |
| Q1: | 0.35±0.046 | 0.99±0.013 | 0.40±0.077 |
| Q4: | 0.34±0.039 | 1.00±0.000 | 0.47±0.090 |
| Non-Embeddable: |  |  |  |
| Quarter | α | λ | τ |
| Q1: | 0.35±0.057 | 0.99±0.009 | 0.47±0.088 |
| Q4: | 0.33±0.051 | 1.00±0.000 | 0.47±0.088 |
| **Dyna-Q** |  |  |  |
| Embeddable: |  |  |  |
| Quarter | α | τ |  |
| Q1: | 0.12±0.063 | 0.30±0.076 |  |
| Q4: | 0.22±0.071 | 0.29±0.061 |  |
| Non-Embeddable: |  |  |  |
| Quarter | α | τ |  |
| Q1: | 0.17±0.086 | 0.27±0.080 |  |
| Q4: | 0.27±0.079 | 0.24±0.078 |  |
| **Explore/Exploit** |  |  |  |
| Embeddable: |  |  |  |
| Quarter | EE |  |  |
| Q1: | 0.20±0.061 |  |  |
| Q4: | 0.05±0.027 |  |  |
| Non-Embeddable: |  |  |  |
| Quarter | EE |  |  |
| Q1: | 0.25±0.079 |  |  |
| Q4: | 0.09±0.061 |  |  |

The model fit parameters were each subject to a two-way repeated measures ANOVA with the predictors condition (embeddable, or non-embeddable) and episode block (first or last quarter). Results revealed no main effects or interactions (all p>0.05) for any of the parameters, except for the Explore/Exploit model, where we found a significant main effect of Episode Block (F(1,14) = 17.80, p = 8.58×10^-4^, η^2^_p_ = 0.56), such that the parameter values for the first quarter of episodes were significantly higher than those for the last quarter.

## ISI Experiment

Parameter values for the different model fits split between the first and last quarters of episodes are given in Table 18 below.

Table 22. ISI experiment parameter values.

| **Sarsa(λ)** |  |  |  |
| --- | --- | --- | --- |
| ISI = 0.5 s |  |  |  |
| Quarter | α | λ | τ |
| Q1: | 0.56±0.06 | 0.99±0.00 | 0.25±0.05 |
| Q4: | 0.63±0.06 | 0.99±0.00 | 0.31±0.06 |
| ISI = 2 s |  |  |  |
| Quarter | α | λ | τ |
| Q1: | 0.72±0.05 | 1.00±0.00 | 0.24±0.07 |
| Q4: | 0.82±0.04 | 0.98±0.02 | 0.26±0.06 |
| ISI = 8 s |  |  |  |
| Quarter | α | λ | τ |
| Q1: | 0.49±0.05 | 1.00±0.00 | 0.18±0.04 |
| Q4: | 0.63±0.05 | 1.00±0.00 | 0.36±0.06 |
|  | | | |
| **Dyna-Q** |  |  |  |
| ISI = 0.5 s |  |  |  |
| Quarter | α | τ |  |
| Q1: | 0.16±0.05 | 0.29±0.08 |  |
| Q4: | 0.25±0.07 | 0.18±0.06 |  |
| ISI = 2 s |  |  |  |
| Quarter | α | τ |  |
| Q1: | 0.16±0.05 | 0.29±0.06 |  |
| Q4: | 0.16±0.06 | 0.12±0.04 |  |
| ISI = 8 s |  |  |  |
| Quarter | α | τ |  |
| Q1: | 0.09±0.05 | 0.31±0.08 |  |
| Q4: | 0.19±0.05 | 0.16±0.05 |  |
|  | | | |
| **Explore/Exploit** | |  |  |
| ISI = 0.5 s |  |  |  |
| Quarter | EE |  |  |
| Q1: | 0.27±0.08 |  |  |
| Q4: | 0.07±0.03 |  |  |
| ISI = 2 s |  |  |  |
| Quarter | EE |  |  |
| Q1: | 0.40±0.09 |  |  |
| Q4: | 0.03±0.01 |  |  |
| ISI = 8 s |  |  |  |
| Quarter | EE |  |  |
| Q1: | 0.36±0.08 |  |  |
| Q4: | 0.06±0.03 |  |  |

The parameter values were subject to two way ANOVAs with factors ISI (0.5 s, 2 s, or 8 s), and quarter (first, last). For Sarsa(λ) there were significant main effects of ISI and quarter on α (ISI: F(2,38) = 9.31, p = 5.11×10^-4^, Quarter: F(1,19) = 8.40, p = 0.009), but no significant interaction (F(2,38) = 0.44, p = 0.646). α-values were higher for the 2 s ISI than for the 0.5 s or 8 s ISI (both p<0.05), but the 0.5 s ISI was not significantly different from the 8 s ISI (p>0.05), and the results from the last quarter had higher α-values than the first quarter at all ISI’s (all p<0.05). For λ and τ there were no significant main effects or interactions (all p<0.05). For Dyna-Q there were no significant main effects or interactions for α. For τ there was a significant main effect of quarter (F(1,19) = 12.25, p = 0.002) such that the last quarter had lower τ-values than the first quarter. There was no main effect of ISI (F(2,38) = 0.3, p = 0.74), and no ISI × Quarter interactions (F(2,38) = 0.27, p = 0.77). For the Exploration/Exploitation model, there was a significant main effect of Quarter (F(1,19) = 25.27, p = 7.49×10-5), but no main effect of ISI and no ISI × Quarter interaction (both p>0.05). The parameter values for the first quarter were higher than for the last quarter (p<0.05).

## Reward experiment

| **Sarsa(λ)** |  |  |  |
| --- | --- | --- | --- |
| Unmotivated |  |  |  |
| Quarter | α | λ | τ |
| Q1: | 0.31±0.04 | 0.99±0.01 | 0.32±0.09 |
| Q4: | 0.33±0.04 | 1.00±0.00 | 0.37±0.08 |
|  |  |  |  |
| Motivated |  |  |  |
| Quarter | α | λ | τ |
| Q1: | 0.35±0.05 | 0.99±0.01 | 0.43±0.08 |
| Q4: | 0.35±0.04 | 1.00±0.00 | 0.37±0.07 |
|  |  |  |  |
| **Dyna-Q** |  |  |  |
| Unmotivated |  |  |  |
| Quarter | α | τ |  |
| Q1: | 0.10±0.05 | 0.30±0.08 |  |
| Q4: | 0.20±0.07 | 0.19±0.06 |  |
|  |  |  |  |
| Motivated |  |  |  |
| Quarter | α | τ |  |
| Q1: | 0.20±0.07 | 0.26±0.08 |  |
| Q4: | 0.22±0.06 | 0.16±0.04 |  |
|  |  |  |  |
| **Explore/Exploit** |  |  |  |
| Unmotivated |  |  |  |
| Quarter | EE |  |  |
| Q1: | 0.32±0.09 |  |  |
| Q4: | 0.33±0.08 |  |  |
|  |  |  |  |
| Motivated |  |  |  |
| Quarter | EE |  |  |
| Q1: | 0.31±0.10 |  |  |
| Q4: | 0.36±0.09 |  |  |

For the reward experiment we conducted 2 × 2 repeated measures ANOVAs on all the parameters with the dependent variables: Motivational condition (unmotivated, motivated), and Quarter (first, last). For Sarsa(λ) we found no significant main effects or interactions for any of the parameters (all p>0.05). For Dyna-Q, there were no significant main effects or interactions for the α parameter (all p>0.05), but we did find a significant main effect of Quarter on the τ parameter (F(1,14) = 5.11, p = 0.040, η_p_^2^ = 0.267), such that the parameter fits for the first quarter were higher than for the last quarter (implying less exploration with increasing time). The remaining effects for τ were not significant (all p>0.05). For the Explore/Exploit model, we found no significant main effects or interactions (all p>0.05).

## Appendix A.7: Successor Representation learning algorithm

Initialize *Q*(*s*; *a*) arbitrarily and *e*(*s*; *a*)= 0, for all *s*, *a*

Initialize M(*s*; *a*; *s*; *a*) = 0, for all *s*, *a*

For each episode:

Initialize *s_old_*, *a_old_*

While *s_old_* is not a goal state:

Take action *a_old_*, observe reward *r* and state *s_new_*

For each possible action a_i_:

$$q\left( i \right)\leftarrow\frac{exp\left( \frac{Q\left( s_{new},a_{i} \right)}{\tau} \right)}{\sum_{j} exp\left( \frac{Q\left( s_{new},a_{j} \right)}{\tau} \right)}$$

End for loop.

Choose *a_new_* from the subject’s actions (normally the algorithm would choose *a_new_* by randomly sampling from the distribution defined by *q*)

The likelihood of *a_new_* is given by:

$L=ln q\left( a_{chosen} \right)+\sum ln\left( 1-q\left( a_{not chosen} \right) \right)$

Update parameters:

$e\left( s_{old},a_{old} \right)\leftarrow e\left( s_{old},a_{old} \right)+1$

For s_i_ ∈ s

For a_i_ ∈ a

For s_j_ ∈ s

For a_j_ ∈ a

If s_New_=s_j_

$\delta\leftarrow1+\gamma M\left( s_{New},a_{New},s_{j},a_{j} \right)-M\left( s_{Old},a_{Old},s_{j},a_{j} \right)$

Else

$\delta\leftarrow\gamma M\left( s_{New},a_{New},s_{j},a_{j} \right)-M\left( s_{Old},a_{Old},s_{j},a_{j} \right)$

End

$M\left( s_{i},a_{i},s_{j},a_{j} \right)\leftarrow M\left( s_{i},a_{i},s_{j},a_{j} \right)+\alpha\cdot\delta\cdot e\left( s_{i},a_{i} \right)$

End

End

End

End

For all *s*, *a*:

$Q\left( s,a \right)\leftarrow Q\left( s,a \right)+M\left( s_{Old},a_{Old},s,a \right)$

$e\left( s,a \right)\leftarrow\gamma\cdot\lambda\cdot e\left( s,a \right)$

End for loop.

$s_{old}\leftarrow s_{new}$

$a_{old}\leftarrow a_{new}$

End while loop.

End for loop.

## Appendix A.8: Hybrid (Sarsa(λ) & Dyna-Q) learning algorithm

Initialize *Q*(*s*; *a*), Q_SL_(*s*; *a*) and Q_DQ_(*s*; *a*) arbitrarily, and *e*(*s*; *a*)= 0, for all *s*, *a*

Initialize environment model M(*s*; *a*; 1:2) = 0 for all *s*, *a*

For each episode:

Initialize *s_old_*, *a_old_*

While *s_old_* is not a goal state:

Take action *a_old_*, observe reward *r* and state *s_new_*

For each possible action a_i_:

$$q\left( i \right)\leftarrow\frac{exp\left( \frac{Q\left( s_{new},a_{i} \right)}{\tau} \right)}{\sum_{j} exp\left( \frac{Q\left( s_{new},a_{j} \right)}{\tau} \right)}$$

End for loop.

Choose *a_new_* from the subject’s actions (normally the algorithm would choose *a_new_* by randomly sampling from the distribution defined by *q*)

The likelihood of *a_new_* is given by:

$L=ln q\left( a_{chosen} \right)+\sum ln\left( 1-q\left( a_{not chosen} \right) \right)$

Update Dyna-Q parameters:

$$\delta_{DQ}=r\left( s_{new},a_{new} \right)+\gamma\cdot\max_{a_{i}} Q_{DQ}\left( s_{new},a_{i} \right)-Q_{DQ}\left( s_{old},a_{old} \right)$$

$$Q_{DQ}\left( s_{old},a_{old} \right)=Q_{DQ}\left( s_{old},a_{old} \right)+\alpha\cdot\delta_{DQ}$$

$$M\left( s_{old},a_{old},1 \right)=s_{new}$$

$$M\left( s_{old},a_{old},2 \right)=r\left( s_{new},a_{new} \right)$$

Dyna-Q Planning:

For *n* planning iterations

Randomly choose a previously visited state s_prev_ and previously performed action at that state a_prev_

$$s_{new simulation}=M\left( s_{prev},a_{prev},1 \right)$$

$$r_{new simulation}=M\left( s_{prev},a_{prev},2 \right)$$

Update Q_DQ_:

$$Q_{DQ}\left( s_{prev},a_{prev} \right)=Q_{DQ}\left( s_{prev},a_{prev} \right)+\alpha\left( r_{new simulation}+\gamma\cdot\max_{a_{i}} Q_{DQ}\left( s_{new simulation},a_{i} \right)-Q_{DQ}\left( s_{prev},a_{prev} \right) \right)$$

End for loop

Sarsa(λ) update:

$$\delta_{SL}=r\left( s_{new}{,a}_{new} \right)+\gamma\cdot Q_{SL}\left( s_{new}{,a}_{new} \right)-Q_{SL}\left( s_{old},a_{old} \right)$$

$e\left( s_{old},a_{old} \right)\leftarrow e\left( s_{old},a_{old} \right)+1$

$$Q_{SL}\leftarrow Q_{SL}+\alpha\cdot\delta\cdot e$$

$$e\leftarrow\gamma\cdot\lambda\cdot e$$

Hybrid Q combination

$Q=w\cdot Q_{SL}+\left( 1-w \right)\cdot Q_{DQ}$

$s_{old}\leftarrow s_{new}$

$a_{old}\leftarrow a_{new}$

End while loop.

End for loop.
